# Comparison of Standard and Free-Hand Region-of-Interest Measurements in Strain Elastography for Differentiating Benign and Malignant Breast Masses

**DOI:** 10.7759/cureus.101363

**Published:** 2026-01-12

**Authors:** Abdulkadir Eren, Emrah Karatay, Irmak Durur-Subasi

**Affiliations:** 1 Radiology, Medipol Mega University Hospital, Istanbul, TUR; 2 Radiology, Sultan II Abdulhamid Han Training and Research Hospital, Istanbul, TUR; 3 Radiology, Istanbul Medipol University, Istanbul, TUR

**Keywords:** benign, breast lesion, breast mass, malignant, region-of-interest, strain elastography

## Abstract

Introduction

Breast cancer remains a prevalent malignancy globally, necessitating accurate and early diagnostic modalities for improved patient outcomes. Strain elastography (SE) is the first and most frequently used elastographic method developed for breast tumor evaluation. SE is widely utilized due to its real-time capabilities and its ability to provide qualitative and semi-quantitative stiffness information.

Objective

This study aimed to compare the diagnostic efficacy of standard region-of-interest (ROI) strain ratio (SR) measurements (circular SR), qualitative Itoh score, and free-hand ROI (all SR) measurements in elastography for distinguishing between benign and malignant breast masses.

Methods

A total of 107 breast masses (55 benign, 52 malignant), all histologically confirmed, were analyzed. Measurements included circular SR (standardized), all SR (operator-defined, free-hand), and the Itoh score (qualitative). Receiver operating characteristic (ROC) curve analysis was used to compare diagnostic performances. Demographic and lesion parameters (age, size, and depth) were analyzed using Mann-Whitney U and chi-square tests.

Results

Both circular SR and all SR measurements were significantly higher in malignant masses (p < 0.0001). Circular SR showed a marginally superior overall diagnostic accuracy with an area under the curve (AUC) of 0.843 (sensitivity: 86.5%, specificity: 76.4%, cut-off > 3.30). The all SR had an AUC of 0.822 (sensitivity: 88.5%, specificity: 74.5%, cut-off > 3.20). The qualitative Itoh score was also a highly significant predictor of malignancy (p< 0.0001). Patients with malignant masses were significantly older (p = 0.0008), but lesion size (p = 0.0596) and depth (p = 0.6090) were not significant differentiating factors.

Conclusion

The standard ROI (circular SR) provides a slightly higher overall diagnostic accuracy, primarily driven by improved specificity. However, the free-hand ROI (all SR) offers higher sensitivity, which is critical in cancer screening. Both quantitative methods, alongside the qualitative Itoh score, are powerful tools for enhancing the diagnostic confidence in breast lesion characterization.

## Introduction

Breast cancer remains a prevalent malignancy globally, necessitating accurate and early diagnostic modalities for improved patient outcomes [1]. This disease is the most common malignancy among women, causing approximately 2.5 million new cases worldwide each year and constituting a significant public health problem [2]. Current data show that one in eight women will develop breast cancer during their lifetime [1,2]. Increasing incidence rates have led to the need for more effective and efficient detection methods. Breast cancer imaging forms the basis of breast cancer screening, diagnosis, preoperative/treatment evaluation, and follow-up. Advanced screening and diagnostic techniques are crucial for early diagnosis, prior to tumor progression or metastasis [3]. Although B-mode ultrasonography (US) and mammography are the main methods used to screen for and diagnose breast cancer, these conventional imaging techniques are not always able to differentiate between benign and malignant tumors, which frequently results in ambiguous results and needless biopsies. Early therapy enhances quality of life, extends patient longevity, and dramatically raises cure rates [4]. While conventional US plays a crucial role in initial assessment, its diagnostic performance can be further enhanced by incorporating advanced techniques such as US elastography [4,5].

Ultrasound elastography, by assessing tissue stiffness, offers valuable insights into differentiating benign from malignant lesions, which often exhibit distinct mechanical properties [1-5]. Among the various elastography techniques, such as strain elastography (SE) and shear-wave elastography (SWE), SE is widely utilized due to its real-time capabilities and its ability to provide qualitative and semi-quantitative stiffness information [2-5]. SE is the first and most frequently used elastographic method developed for breast tumor evaluation. Based on the idea that malignant tumors typically show more stiffness than benign tissues because of variations in tissue architecture, cell composition, and extracellular matrix deposition, this technique offers diagnostic support [6]. SE is usually performed with the same superficial linear ultrasound probe used for B-mode. This method estimates tissue stiffness based on tissue displacement caused by external manual compression or patient-induced movements (physiological movements of the heart and respiration). It then converts the displacement gradient into pixels for imaging, providing semi-quantitative data [7]. In clinical practice, SE is used together with US as an auxiliary tool to further characterize breast lesions. SE may lessen the amount of needless breast biopsies and is helpful in distinguishing benign from malignant masses. Thus, it is quite useful as a potential imaging biomarker [6,7]. Lesion hardness can be displayed on a color scale for qualitative assessment or expressed as a fat-to-lesion strain ratio (SR) for semi-quantitative assessment. When evaluating using the qualitative method (Itoh score), the strain distribution is superimposed on the standard B-mode US image to create a color-coded map [8]. The original B-mode US image is shown next to the resulting combined B-mode and strain image [5-8].

However, the diagnostic accuracy of SE is highly dependent on the proper selection and placement of the region of interest (ROI). The choice between a standard (fixed) ROI and a free-hand (operator-defined) ROI can significantly impact the calculated strain ratios and, consequently, the diagnostic performance and interobserver variability, particularly given the operator-dependent nature of SE [9]. The resulting SE image is also affected by the individual performer's compression technique. This technique depends on the deformability of the organ and the skill of the operator, which can significantly affect the images. Interobserver variability during image acquisition and interpretation may also partially limit the use of SE in routine clinical practice. When used effectively, this technique can reduce the number of unnecessary breast biopsies [7,9].

This study aimed to compare the diagnostic efficacy of standard and free-hand ROI measurements in SE for distinguishing between benign and malignant breast masses. The qualitative Itoh score was also calculated, and its effectiveness in the diagnosis of malignancy was evaluated.

## Materials and methods

Study design and population

This study was approved by the local ethics committee of the Istanbul Medipol University School of Medicine (Approval No.: E-10940078-2025.4174-785). The 1964 Helsinki Declaration was fully complied with throughout the study, and all patients evaluated gave written informed consent. A retrospective archive search of patients who underwent breast ultrasound in the radiology unit between February 2020 and October 2025 was conducted. Patients with a history of chest trauma, age < 18 years, those who had previously undergone breast surgery, those who received radiotherapy and/or chemotherapy for any reason, and those diagnosed with granulomatous mastitis were excluded from the study. A total of 107 breast masses that underwent strain elastography examination were included in the study. All masses were confirmed by final histopathological diagnosis (55 benign, 52 malignant), and all included cases had a single breast lesion. The strain values ​​of the cases with biopsy results and those in which the SE technique was applied were compared. Baseline characteristics collected included patient age, average lesion size (mm), and lesion depth category.

Strain elastography measurements

A radiologist with almost 10 years of experience in breast radiology carried out the SE procedures. Whole elastography examinations were performed using an ultrasound system (Logiq E9 XDclear 2.0, GE Healthcare, Chicago, IL) with a linear array probe (ML6-15 MHz). All patients were evaluated in the supine position, and both breasts and axillary fossae were scanned in the longitudinal and transverse planes. The Young modulus principle was used to determine the strain in the axial dimension during SE measurements. This aimed to visualize the degree of compression or extension following tissue deformation. SE images and strain values ​​were obtained as a result of repeated manual compression of the US probe [10]. To obtain optimal SE measurements, images were selected from the decompression phase, and strain values ​​were calculated for each lesion by the radiologist. SE images were taken by applying gentle pressure on the breast tissue via the transducer to ensure proper contact with the skin, causing the breast tissue to displace posteriorly by 1-2 mm, thus returning to its initial position. The elastographic evaluation was performed using standard ROI and the free-hand ROI techniques [11]. Both qualitative and quantitative measurements were recorded.

Itoh Score (Qualitative)

Lesions were assigned an elasticity score from 1 to 5 based on the visual pattern of stiffness (typically 1-2 for benign, and 4-5 for malignant) [8].

Circular SR (Standard ROI)

A fixed, predefined circular ROI was placed at the stiffest part of the lesion, and a reference ROI of the same size was placed in the adjacent fat tissue at the same depth. The SR was calculated automatically by the system. This method prioritizes reproducibility [9,10].

All SR (Free-Hand ROI)

**Figure 1:**
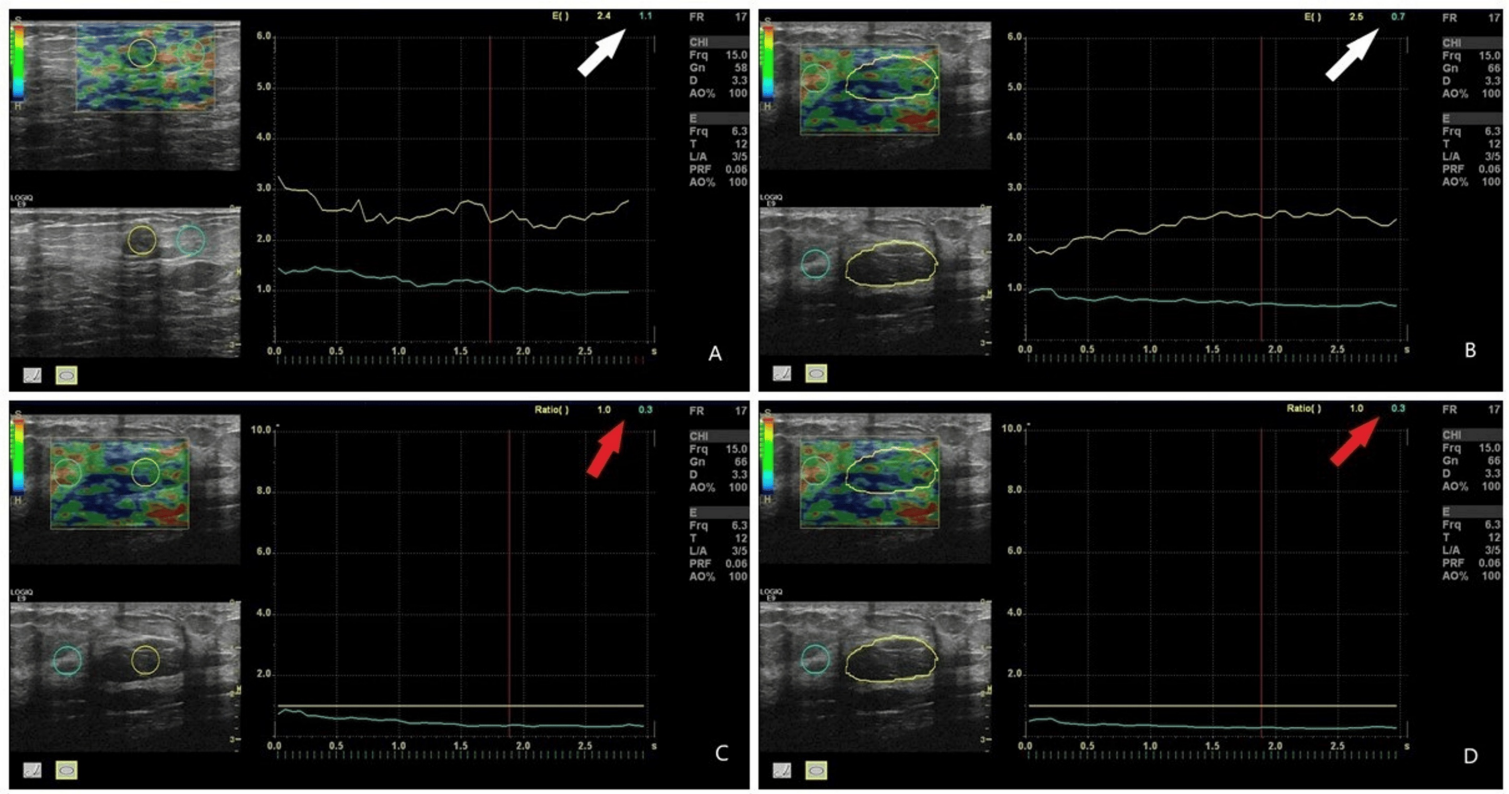
A 47-year-old female patient has a fibrocystic pattern in her breast, and images from strain elastography are available. The green ROI was placed in the fatty tissue, and the yellow ROI in the target tissue. In panel A, the standard ROI and in panel B, the free-hand ROI are used to calculate the Itoh score, with values indicated by a white arrow in the upper right corner, showing lower values (1-2) supporting a benign pathology. In panels C and D, the same sequence is followed, and the strain ratio values are shown by a red arrow in the upper right corner. ROI: region of interest.

**Figure 2:**
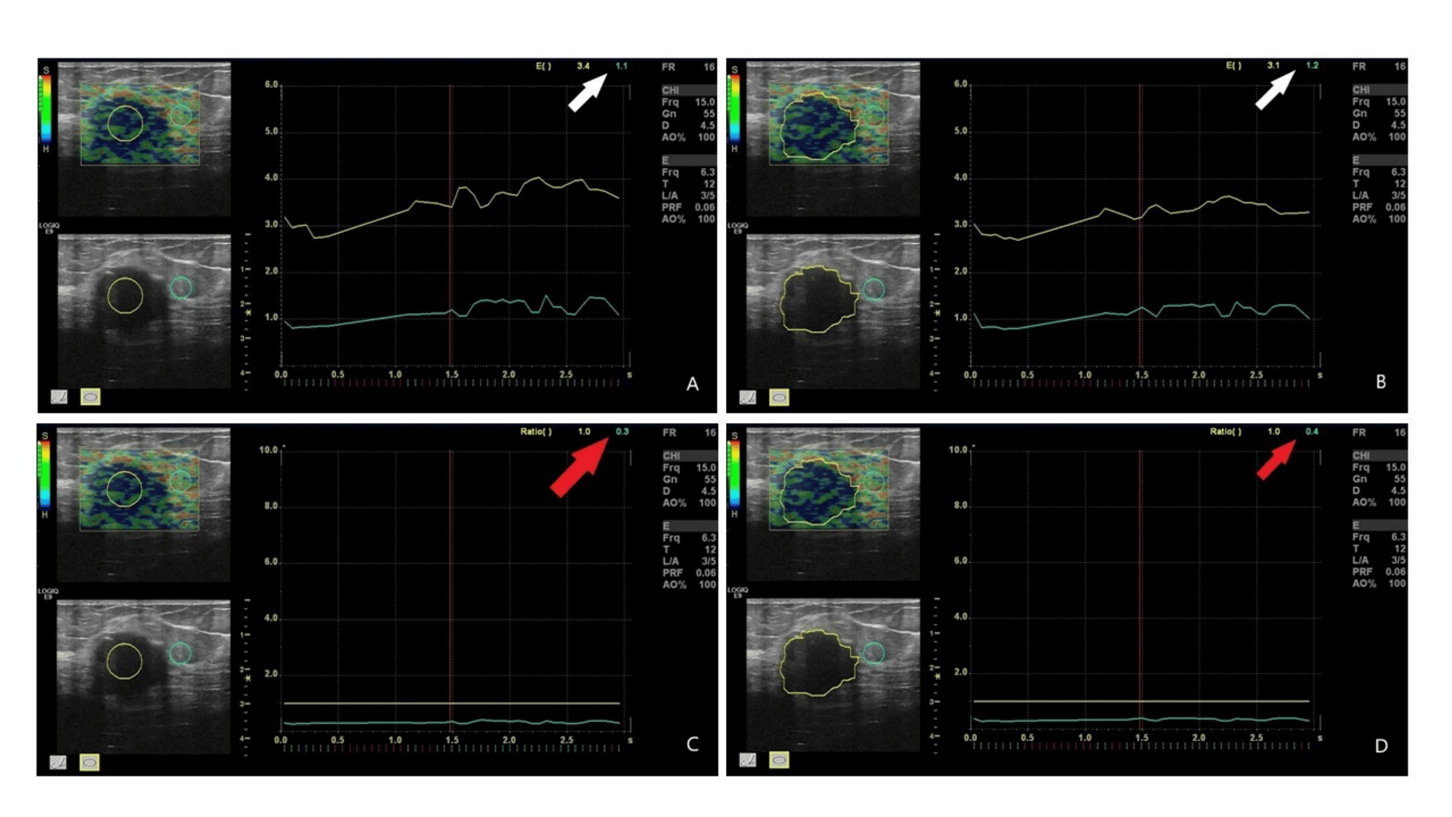
In strain elastography images of a 55-year-old patient diagnosed with invasive ductal carcinoma. The green ROI was placed in fatty tissue, and the yellow ROI in the target tissue. In panel A, the standard ROI and in panel B, the free-hand ROI and Itoh score values are shown with a white arrow in the upper right corner. The Itoh score for malignant lesions obtained with free-hand ROI was lower, while the Itoh score for standard ROI was higher and more specific (3-4-5). The same sequence is followed in panels C and D, and the strain ratio values are shown with a red arrow in the upper right corner. ROI: region of interest.

The operator manually outlined the lesion boundaries (or the entire area of interest) on the elastogram using an irregular, free-hand shape. A reference ROI was placed in the fat tissue. This method aims to capture lesion heterogeneity more precisely [12]. For each lesion, whether benign or malignant, the Itoh score was calculated first, followed by the standard ROI, and finally, all ROI measurements during the measurement process (Figures 1, 2).

Statistical analysis

The data were statistically analyzed using IBM SPSS version 21 (IBM Corp., Armonk, NY). The data distribution was examined using the Kolmogorov-Smirnov test. Descriptive statistical methods (mean, standard deviation, percentage, minimum, and maximum) were used to evaluate and express central tendency. The means of continuous variables (age, lesion size, circular SR, all SR, and Itoh score) between the benign and malignant groups were compared using the Mann-Whitney U test. The relationship between categorical variables (lesion depth category and malignancy) was discovered using the chi-square test. Receiver operating characteristic (ROC) curve analysis was performed to determine the area under the curve (AUC) for circular SR and all SR, establishing optimal cut-off values and their corresponding sensitivity and specificity. A p-value < 0.05 was regarded as statistically significant at a 95% confidence range.

## Results

A total of 107 breast masses were included in the study, 55 of which were benign and 52 were malignant. The mean age of the overall study cohort (n = 107) was 42.61 ± 13.15 years. The youngest case was 23 years old, and the oldest case was 71 years old; all cases were of females. Patients with malignant masses were significantly older (mean age: 46.40 ± 13.29 years) than those with benign masses (mean age: 39.02 ± 12.79 years) (Mann-Whitney U test, p = 0.0008).

The smallest benign breast mass measured 4.56 mm, and the largest was 30.58 mm. The smallest malignant breast mass measured 5.02 mm, and the largest was 28.44 mm. Malignant masses (16.09 ± 5.89 mm) were slightly larger than benign masses (14.01 ± 6.58 mm), but this difference was not statistically significant (p = 0.0596).

The chi-square test revealed no statistically significant association between the lesion depth category and malignancy status (chi^2^ = 2.70, p = 0.6090).

The ratio measurements for the population were carried out using circular SR and all SR, respectively. Both semi-quantitative methods showed a highly significant difference between the benign and malignant groups (p < 0.0001 for both). A comparison of tension levels between benign and malignant groups is summarized in Table 1.

**Table 1:** Comparison of strain ratio measurements between benign and malignant masses. ROI: region of interest; SR: strain ratio; n: number; SD: standard deviation.

Strain ratio measurement	Malignancy status	Number of lesions (n)	Mean ± SD	Mann-Whitney U p-value
Circular SR (Standard ROI)	Benign	55	2.78 ± 1.35	p < 0.0001
	Malignant	52	4.88 ± 1.83	
All SR (Free-Hand/Entire ROI)	Benign	55	2.83 ± 1.25	p < 0.0001
	Malignant	52	4.71 ± 1.76	

The circular SR (standard ROI) method yielded a marginally superior AUC of 0.843, while the all SR (free-hand/entire ROI) method yielded an AUC of 0.822.

The all SR method demonstrated a slightly higher sensitivity (88.5%) while the circular SR method provided a slightly higher specificity (76.4%) (Table 2).

**Table 2:** Diagnostic performance of standard and free-hand strain ratio measurements. AUC: area under the curve; ROI: region of interest; SR: strain ratio.

Strain ratio measurement	AUC	Optimal cut-off value	Sensitivity	Specificity
Circular SR (Standard ROI)	0.843	>3.30	86.5%	76.4%
All SR (Free-Hand/Entire ROI)	0.822	>3.20	88.5%	74.5%

The qualitative Itoh score was also a powerful predictor of malignancy. The mean Itoh score was significantly higher for malignant masses (3.56 ± 0.94) compared to benign masses (2.38 ± 0.83) (p < 0.0001). The distribution of malignant and benign lesions according to Itoh scores is shown in Table 3. The distribution showed a clear shift toward higher scores (4 and 5) in the malignant group, confirming its diagnostic utility.

**Table 3:** Distribution of Itoh scores by benign and malignancy status.

Itoh score	Benign (0)	Malignant (1)
1	5	0
2	30	7
3	15	18
4	4	18
5	1	9

## Discussion

One of the main causes of cancer-related mortality, breast cancer is the most prevalent type of cancer diagnosed in women globally. Breast cancer is the most frequent cancer among women in North America and the second most common cause of cancer-related deaths, after lung cancer [13]. Treatment and survival depend on an early diagnosis. Individuals who are diagnosed with smaller primary breast cancers have a much higher chance of surviving and a significantly reduced chance of dying from the disease [14]. An increasingly used technique for describing localized lesions in the breast is ultrasound elastography. Various techniques (SE, SWE, etc.) and image analyses can be used for characterization. Elastography is increasingly used in the clinical diagnosis of breast tumors due to its ease of use and diagnostic potential when used in conjunction with B-mode US. SE and SWE are frequently recommended in daily practice for assessing the stiffness of breast tumors [15]. SE is a semi-quantitative method in which tumor tension is ratioed to the tension of the surrounding tissue. Tension is shown as a color code superimposed on the B-mode image and is inversely correlated with texture stiffness [16].

Our results confirm that both the standardized circular SR and the free-hand all SR methods are robust quantitative markers for differentiating benign from malignant breast masses, both displaying excellent diagnostic utility (AUC > 0.82) [17].

The circular SR (standard ROI) method demonstrated a marginally superior overall diagnostic accuracy (AUC: 0.843). This slight advantage was primarily driven by its higher specificity (76.4% vs. 74.5% for all SR). This suggests that the standardized placement of the ROI, especially the external reference ROI in the adjacent fat tissue, may yield more reproducible results and effectively reduce the inclusion of non-stiff surrounding tissue, thereby lowering the rate of false-positive diagnoses [18].

Conversely, the all SR (free-hand/entire ROI) method achieved a higher sensitivity (88.5% vs. 86.5% for circular SR). This finding indicates that tracing the full extent of the lesion on the elastogram is highly effective in capturing the stiffness indicative of malignancy, potentially minimizing false-negative results, which is critical in cancer screening.

The qualitative Itoh score was found to be a highly significant predictor of malignancy (p < 0.0001). This underscores that even the visual, operator-dependent assessment of tissue stiffness remains a critical and powerful tool, offering comparable diagnostic power to the quantitative strain ratios. This supports the clinical use of the Itoh score, particularly in high-volume settings where efficiency is paramount [9].

Consistent with established literature, patient age was a significant risk factor for malignancy (p = 0.0008). However, lesion size (p = 0.0596) and lesion depth (p = 0.6090) were not found to be statistically significant differentiating factors. This reinforces the value of tissue elasticity assessment over basic B-mode measurements in characterizing breast masses [19].

Limitations of the study

The study's limitations include its retrospective design and the inherent operator-dependency of strain elastography [9]. While lesion depth was not significant, the low number of cases in the deepest categories (>15 mm) may limit the generalizability of this specific finding. For clinical practice, integrating both ROI methods may optimize diagnostic accuracy: initial screening using the efficient circular SR method, followed by detailed free-hand ROI analysis for suspicious or borderline lesions.

## Conclusions

Both the standard (circular SR) and free-hand (all SR) ROI methods provide high diagnostic utility for differentiating benign and malignant breast masses. The standard ROI method offered marginally superior overall accuracy due to higher specificity, while the free-hand ROI method provided superior sensitivity. Considering the minimal difference in AUC values, the choice of method may depend on institutional priorities (reproducibility vs. capturing heterogeneity). Both quantitative methods, coupled with the highly significant qualitative Itoh score, are essential for improving clinical decision-making and reducing unnecessary invasive procedures.

## References

[1] Yang H, Xu Y, Zhao Y, Yin J, Chen Z, Huang P (2020). The role of tissue elasticity in the differential diagnosis of benign and malignant breast lesions using shear wave elastography. BMC Cancer.

[2] Kim J, Harper A, McCormack V (2025). Global patterns and trends in breast cancer incidence and mortality across 185 countries. Nat Med.

[3] Fujita T (2016). Adjunctive ultrasonography for breast cancer screening. Lancet.

[4] Yoon JH, Song MK, Kim EK (2017). Semi-quantitative strain ratio determined using different measurement methods: comparison of strain ratio values and diagnostic performance using one- versus two-region-of-interest measurement. Ultrasound Med Biol.

[5] Meng W, Zhang G, Wu C, Wu G, Song Y, Lu Z (2011). Preliminary results of acoustic radiation force impulse (ARFI) ultrasound imaging of breast lesions. Ultrasound Med Biol.

[6] Navarro B, Ubeda B, Vallespí M, Wolf C, Casas L, Browne JL (2011). Role of elastography in the assessment of breast lesions: preliminary results. J Ultrasound Med.

[7] Barr RG (2014). Elastography in clinical practice. Radiol Clin North Am.

[8] Itoh A, Ueno E, Tohno E (2006). Breast disease: clinical application of US elastography for diagnosis. Radiology.

[9] Turnaoğlu H, Haberal KM, Arslan S, Yavuz Çolak M, Ulu Öztürk F, Uslu N (2021). Interobserver and intermethod variability in data interpretation of breast strain elastography in suspicious breast lesions. Turk J Med Sci.

[10] Dietrich CF, Barr RG, Farrokh A (2017). Strain elastography - How to do it?. Ultrasound Int Open.

[11] Kanagaraju V, Dhivya B, Devanand B, Maheswaran V (2021). Utility of ultrasound strain elastography to differentiate benign from malignant lesions of the breast. J Med Ultrasound.

[12] Park HJ, Kim SM, Yun B, Jang M, Kim B, Lee SH, Ahn HS (2020). Comparison of one- and two-region of interest strain elastography measurements in the differential diagnosis of breast masses. Korean J Radiol.

[13] Siegel RL, Miller KD, Jemal A (2015). Cancer statistics, 2015. CA Cancer J Clin.

[14] van der Sangen MJ, Poortmans PM, Scheepers SW, Lemaire BM, van Berlo CL, Tjan-Heijnen VC, Voogd AC (2013). Prognosis following local recurrence after breast conserving treatment in young women with early breast cancer. Eur J Surg Oncol.

[15] Carlsen J, Ewertsen C, Sletting S, Vejborg I, Schäfer FK, Cosgrove D, Bachmann Nielsen M (2015). Ultrasound elastography in breast cancer diagnosis. Ultraschall Med.

[16] Mutala TM, Mwango GN, Aywak A, Cioni D, Neri E (2022). Determining the elastography strain ratio cut off value for differentiating benign from malignant breast lesions: systematic review and meta-analysis. Cancer Imaging.

[17] Barr RG, De Silvestri A, Scotti V, Manzoni F, Rebuffi C, Capittini C, Tinelli C (2019). Diagnostic performance and accuracy of the 3 interpreting methods of breast strain elastography: a systematic review and meta-analysis. J Ultrasound Med.

[18] Evans A, Whelehan P, Thomson K (2010). Quantitative shear wave ultrasound elastography: initial experience in solid breast masses. Breast Cancer Res.

[19] Yılmaz E, Yılmaz A, Aslan A, Inan I, Evren MC, Tekesin K (2017). Real-time elastography for differentiation of breast lesions. Pol J Radiol.

